# Can Post-Retirement Work Always Prevent Depression?

**DOI:** 10.3390/bs13060466

**Published:** 2023-06-03

**Authors:** Haiting Yan, Juan Liu, Wei Wei, Hongyan Xu, Xu Huang, Jiaxin Ying

**Affiliations:** 1 School of Digital Economics and Management, Wuxi University, Wuxi 214105, China; 2 Rosen College of Hospitality Management, University of Central Florida, Orlando, FL 32819, USA

**Keywords:** post-retirement work, depression, adaptation ability, conditional factors, China

## Abstract

Given the rising popularity of post-retirement work and its potential benefits for older adults’ mental health, this study examined older adults’ adaptation ability as a conditional factor for the impact of post-retirement work on depression. Quantitative data from 1433 working older adults and 1433 non-working older adults were analyzed using the PROCESS macro in SPSS to test a moderated regression model with adaptation ability as the moderator. Results showed that older adults with lower adaptation ability demonstrated significantly lower depression if they worked (vs. did not work). Older adults with higher adaptation ability exhibited significantly higher depression if they worked (vs. did not work). These findings were subsequently verified with a robustness check. Overall, post-retirement work did not prevent depression for the entire sample; working only alleviated depression among older adults with limited adaptation ability. Older adults with stronger adaptation ability could better maintain their mental health by staying retired. This study fills a knowledge gap regarding the relationship between post-retirement work and mental health. Implications for aging societies are also discussed.

## 1. Introduction

The aging of the population accompanies the decline of the working-age population [1] which presents a financial pressure on governments and societies [2]. To mitigate this financial pressure, many countries have advocated post-retirement work [3]. Post-retirement work refers to any form of paid work that takes place after the individual’s retirement or retirement age [4]. With the loss of work functions including financial resources, social interactions, and even self-identity, retirement is a major life event and significant challenge for older adults [5]. Post-retirement work however may remedy these losses [6] and thus smooth retirement transition [7]. While many countries and regions, such as the U.S. [8], European Union [9], China [10], Singapore [11], and Japan [12], either have raised the full retirement age or are planning to do so as a formal policy [10], post-retirement work has already gained its popularity among older adults in many societies. For instance, 1.5 million of American near-retirees are willing to go back to work [13]; an increase in post-retirement work is also witnessed in EU countries [14]; 64% of the Japanese population is willing to work beyond retirement age [15]; post-retirement work in China with the most elderly population in the world is also recognized as a certain trend [3].

In addition to fulfilling older adults’ financial need, post-retirement work is found to benefit the health of older adults, including preventing depression [16,17]. Depression is defined as a psychiatric disorder characterized by depressed mood, diminished energy, lack of concentration, low self-esteem, and disturbed sleep [18]. As older adults are at an increased risk for experiencing depression [19], it might be worthwhile for older adults to prevent depression by engaging in post-retirement work. Nevertheless, research regarding the impact of post-retirement work on depression is not sufficient. Specifically, research concerning conditional factors that may affect post-retirement work’s influence on depression is very scarce. Investigating the conditional factors may clarify when post-retirement work can or cannot prevent depression. In such, post-retirement work can be better designed and thereby assure its benefits for older adults’ mental health.

The dramatic process of social change and technology development in the 21st century presents challenges and stresses to older adults. In addition to these macro-level social and technological challenges, micro-level challenges are present for older adults as well. For instance, for older workers who engage in non-career jobs, they may have to learn new skills and build new social networks. Even for those who stay in their career jobs, they may face challenges such as ageism, concerns of stealing jobs or blocking promotion for younger generations, gossip of why they do not enjoy later life with more leisure, etc. [20]. For older adults who stay in retirement, the loss of financial resources, social interactions, self-identity, etc., are significant life changes and dramatic challenges [21]. Older adults’ adaptation to these changes determines their life quality and well-being [22]. Therefore, no matter working or not, post-retirement life requires older adults’ adaptation ability. Given the important role of adaptation ability in older adult’s life and work, it is valuable to examine older adult’s adaptation ability as a conditional factor. The purpose of this study was thus to test the moderating effect of adaptation ability in the association between post-retirement work and depression. The results of this study will make the following contributions: (1) to enrich the literature in the association between post-retirement work and depression, (2) to illustrate the impact of post-retirement work on depression among older adults with different levels of adaptation ability, and (3) to provide suggestions regarding how to prevent depression through post-retirement work. 

## 2. Literature Review

### 2.1. Antecedents and Outcomes of Post-Retirement Work

Older adults pursue post-retirement work for various reasons. In addition to meeting financial need, older adults expect social, personal, and generative rewards from work [23], such as a social network [20], self-actualization [24], mentoring young generations [25], and staying physically and mentally active [26].

Older adults’ pursuit of post-retirement work is associated with four categories of antecedents: individual attributes, family-related variables, job-related variables [27], and macro factors [28]. Specifically, for individual attributes, younger age [29], higher level of education [30], better health status [31], and lower financial status [32] are the individual drivers for post-retirement employment. An individual’s aging experience also influences an older adult’s decision on post-retirement employment [33]. For family-related variables, older adults who have working spouse and/or dependent children [29] are more likely to work after retirement. Job-related variables include both preretirement job-related variables and potential post-retirement job-related variables. Older adults with higher job satisfaction with their pre-retirement jobs are more likely to pursue work after retirement age [27]. Jobs that provide fair treatment, age-supportive management policies, and flexible work opportunities are more attractive to older adults [34]. Macro factors such as labor market demand and policy support are also impetuses for post-retirement employment [35].

Post-retirement work has been found to bring favorable outcomes for older adults. For example, financial stability for later life is apparent [4]. As post-retirement work offers the opportunity for older adults to maintain active, productive, and social interactions, it benefits both their physical and mental health [17,36,37] and, consequently, increases their life satisfaction [38]. The beneficial impact of post-retirement work is explained by the continuity theory [39] and the role theory [40]. The continuity theory suggests that individuals could maintain or increase their level of well-being if they can continue their past daily routines. The role theory emphasizes the role transition in retirement which is a process of losing work roles. This process of role loss may cause older adults’ feelings of anxiety and depression, while post-retirement work can smooth this transition [41]. Yet, some studies reported that experience and outcome of post-retirement work varied according to pre-retirement context and socio-demographic attributes of retirees, as well as cultural experience of aging in different communities. Contrary to most of the studies in western countries, Xie et al. (2021)’s research using Chinese sample reported that post-retirement employment significantly increased Chinese older adults’ depression scores and worsened their mental health, and this negative impact is stronger for those who are older and female, with a lower educational level, higher pensions, higher social status, and living in urban areas [42]. Dinegemen and Henkes (2014) showed that post-retirement work could mitigate the decreased life satisfaction of involuntary retirees and enhance the well-being of voluntary retirees [43]. Dinegemen and Henkes (2015) indicated that post-retirement work may increase life satisfaction of older adults who work with intrinsic motives but in contrast may decrease life satisfaction of those who work with financial motives [44]. Lux and Scherger (2018) posited that post-retirement work could not benefit older workers’ health if they work in a different field other than preretirement job [45]. These mixed findings suggest to further investigate: (1) the impact of post-retirement work on mental health in different cultural context and (2) the underlying relationship between post-retirement work and older adults’ mental health by identifying conditional factors. The present study aims to fill the gap by examining the impact of post-retirement work on depression among Chinese older adults and the moderating effect of older adult’s adaptation ability in the link between post-retirement work and depression.

With the foundational support of continuity theory, role theory, and the majority studies that have demonstrated positive impact of post-retirement work on mental health, we hypothesize that:

**H1:**  *Post-retirement work is negatively associated with depression*.

### 2.2. Theoretical Foundations for the Moderating Role of Adaptation Ability

Although the literature lacks conditional factors for the underlying relationship between post-retirement work and older adults’ mental health, the person–environmental fit theory [46] and the trait activation theory [47] along with the ecological theory of aging [48] unanimously suggest adaptation ability as a conditional factor for post-retirement work’s preventing role in depression. Adaptation ability refers to older adults’ ability to adapt to the environment and their changing roles [49].

According to the person-environmental fit theory, when one fails to fit with environmental demands, one will experience a higher level of stress. The cumulative stress caused by the misfit may impair one’s mental health [50]. If older adults who are not adaptive chose to work, the environmental/organizational demands could increase their stress which may jeopardize their mental health. Based on this theory, Lahlouh et al. (2019) further tested the relationship between older adults’ intention for post-retirement employment and three categories of person–environment fit (needs–supplies, demands–abilities, and Value congruence) [51]. They uncovered that value congruence at vocational level, needs and supplies fit at organizational and job levels, and the fit between older worker’s abilities and job demands were positively related to the intention to pursue post-retirement employment. Although Lahlouh et al. (2019)’s study examined older adults’ intention for post-retirement work rather than their experiences in post-retirement work [51], their findings enlighten the potential influence of the different categories of person–environment fit on older adults’ experiences in post-retirement work. 

The trait activation theory describes trait–situation relevance to explain outcomes [47]. The theory proposes that when an individual’s trait matches cues in a situation, namely the trait and situation relevant, favorable outcomes will result including positive reaction to the situation and better performance. The cumulative favorable reaction and performance would decrease stress and further benefit mental health [52]. As adaptive (trait) older adults match the demand of environmental/organizational change better (situation), they would experience less work stress and better mental health than their counterparts do. Lievens et al. (2018) further pointed out that an individual trait is dependent on different occasions and contexts [53]. That is to say, older adults’ adaptation ability may vary in line with their environment, and as such, employers may be able to adjust older adults’ adaptation ability by changing work environment.

According to the ecological theory of aging, successful aging occurs when one finds the best balance between competence and environmental press. Competence is defined as the theoretical upper limit of capacity of the individual to function in the area of biological health, sensation perception, motoric behavior, and cognition [54]. Environmental press refers to the environmental force from the physical or social perspectives that older adults have to face in their lives [48]. The theory argues that when older adults have more compatible levels of competence and environmental press, they are more likely to exhibit adaptive behavior, which will ultimately result in positive outcomes. In contrast, those with low levels of competence but experiencing excessive environmental pressure may have maladaptive behavior, which in turn may lead to negative consequences (e.g., deteriorating health conditions). In other words, the level of adaptation ability of working older adults may represent their balance level of competence and environmental stress, therefore playing an important role in their working context, and may moderate the impact of post-retirement work on older adults’ health. 

In accordance with the three theories, it is presumable that post-retirement work would benefit the mental health of older adults when they have higher level of adaptation ability but would boost mental stress of older adults who have lower level of adaptation ability. We therefore develop the hypotheses as follows:

**H2:**  *Post-retirement work increases depression in older adults who have a lower level of adaptation ability*.

**H3:**  *Post-retirement work decreases depression in older adults who have a higher level of social adaptation*.

## 3. Methods

### 3.1. Data Source

The hypotheses were tested with the secondary data obtained from the individual survey of the China Longitudinal Aging Social Survey (CLASS) offered by the National Survey Research Center at Renmin University of China. CLASS is a nationwide social survey project conducted in 2016 and collects social, psychosocial, and economic data about Chinese older adults (aged 60 and above) on individual and community levels regularly and systematically. A total of 462 neighborhoods in urban areas and villages in rural areas were randomly selected from 28 of China’s 31 provinces for CLASS. A total of 25 households with older adults were randomly chosen from each selected village or neighborhood. Researchers approached the sampled households, read questions of the survey to one of the older adults in each household, and filled out the survey based on respondents’ answers.

The CLASS datasets have been popularly used by a number of published studies [55,56,57]. The dataset contains a total of 11,494 respondents (1433 working older adults and 10,060 non-working older adults). Since the dataset is unbalanced by post-retirement work, which may bias the results towards the majority class [58], the non-working class in this case, we therefore randomly selected a subset of samples from the non-working class to match the number of working samples to reduce this bias problem [59] by using the random case selection function of SPSS. The final dataset used in the study includes 1433 working and 1433 non-working samples.

The retirement policy in China implements a compulsory retirement age: 60 for men, 55 for female cadres/professionals (including teachers, medical personnel, administrators, and other professionals), and 50 for the rest of the female workers [60]. Once people reach their retirement age, they must leave their work and start to receive pension. For people who were unemployed previously (e.g., farmers and unemployed urban residents), if they have participated in “the endowment insurance system for urban and rural residents” funded by the government, they can start to receive pension once they reach 60 [61]. Given the financial pressure of pension system under the rapid population aging, China has been considering postponing the official retirement age [60]. As a result of the long-time mandatory retirement policy as well as the filial piety culture, retirement for older adults is for enjoying life with support from adult children, and working after retirement may be regarded by the overall Chinese society as having ungrateful children or family misfortune [42]. Studies have shown that financial need is the main driver of post-retirement work for Chinese older adults [62,63].

### 3.2. Measures

Depression was assessed by nine items (Cronbach’s α = 0.73; KMO = 81.3, *p* < 0.001) from the Center for Epidemiologic Studies–Depression scale (CES-D) on a 3-point Likert-type scale (1 = “hardly ever”, 2 = “some of the time”, and 3 = “often”) adapted from Silverstein et al. (2006) [64]. Of the nine items, three addressed positive effects (happiness, enjoyability, and pleasure); two relate to negative effects (loneliness and feeling upset); two concerned feelings of marginalization (feeling useless and having nothing to do), and two reflected somatic symptoms (poor appetite and trouble sleeping). Sample items include: “Do you feel lonely?”, “Do you feel useless?”, and “Do you feel sad?”. A higher score indicates higher level of depression. Post-retirement work was measured by one question: “Are you currently engaged in any paid work?”; the answer was coded as 1 = yes, 0 = no. Eight items of adaptation ability (Cronbach’s α = 0.79; KMO = 80.8, *p* < 0.001) were adopted from Chen (2006) [65] and Chen et al. (2019)’s [66] Social Adaptation Scale (SAS) and rated on a 5-point Likert-type scale (from 1 = not fitting to 5 = fitting) [65,66]. Sample items include “I like to learn”, “The world changes so fast, and I cannot adapt to the change” (reverse coded), and “The changes of the world are more and more unfavorable to older adults” (reverse coded). A higher score presents a higher level of adaptation ability. Age, gender, marital status, educational level, neighborhood type, perceived health status, and pension status were included in the entire process of analyses as control variables, as they have been found to be correlated with post-retirement work [67]. Gender was coded as 1 = male, 0 = female; neighborhood type was coded as 1 = rural, 0 = urban; pension status was assessed by one question: “Do you receive pension?” and was coded as 1 = yes, 0 = no. Health was measured by one item of self-rated health status: “How do you think of your current health status?” Options of their answer include 1 = very unhealthy, 2 = unhealthy, 3 = neutral, 4= healthy, and 5 = very healthy.

### 3.3. Data Analyses

The data analysis process consisted of three steps. Descriptive analysis was performed first to demonstrate characteristics of the study samples with the means and standard deviations of the study variables. Second, a moderated regression model with adaptation ability as a moderator was tested to examine the impact of post-retirement work on depression. The Hayes (2017) SPSS macro PROCESS (http://www.afhayes.com, accessed on 31 January 2023) was executed in SPSS to examine the proposed moderating effect [68]. Third, a robustness check was performed with a new randomly selected dataset from the original full dataset. This dataset used for robust check includes 1433 new randomly selected non-working samples and the original 1433 working samples. 

## 4. Results

### 4.1. Descriptive Statistics

Table 1 summarizes the characteristics of the study sample, the means, and standard deviations of study variables. Our dataset consisted of 1433 working older adults and 1433 non-working older adults. Over 40% of the working group were aged 60–64; the majority of non-working group was evenly distributed from 60 to 74. Slightly more than half of non-working group were female, while nearly 60% of working group were male. Most respondents were married (68.7% for non-working and 82.2% for working). The majority of non-working older adults were either illiterate (22.9%) or had an education at an elementary school level (35.8%). The majority of working older adults had completed elementary school (39.9%) or middle school (28.5%). More than half of both non-working (56.6%) and working group (58.9%) were rural older adults. The health status (3.55 for working group and 3.30 for non-working group, *p* < 0.01) and adaptation ability (2.99 for working group and 2.93 for non-working group, *p* < 0.05) of the working group are significantly higher than non-working group. The depression level of working group is significantly lower than non-working group (1.67 for working group and 1.70 for non-working group, *p* < 0.05). 

### 4.2. Analysis of Moderating Effect

Table 2 displays the results of regression analyses with adaptation ability as a moderator. Post-retirement work significantly prevents depression (B = −0.54, *p* < 0.01), supporting H1, and adaptation ability as an independent variable does not influence depression (B = −0.04, *p* > 0.05). The significant interaction effect of post-retirement work and adaptation ability (B = 0.19, *p* < 0.01) indicates that adaptation ability significantly moderates the impact of post-retirement work on depression.

**Figure 1:**
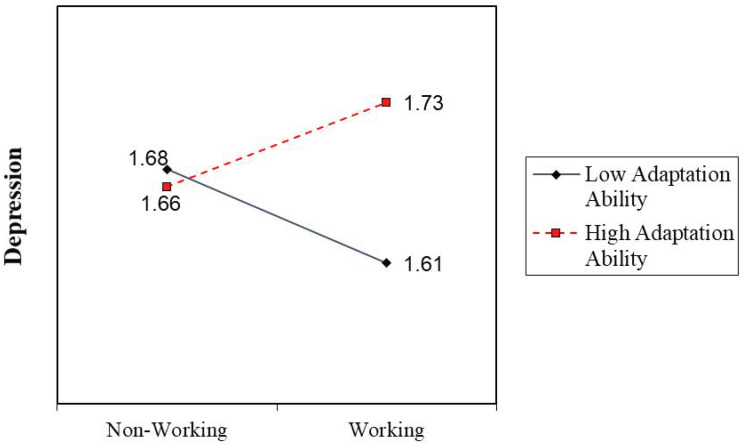
The impact of post-retirement work on depression at high and low levels of adaptation ability.

Table 3 shows the conditional effects of post-retirement work at different levels of adaptation ability. For older adults with lower adaptation ability (1 standard deviation lower from mean), post-retirement work decreases their possibility of depression. This tests but does not support H2. For older adults with an average level of adaptation ability, post-retirement work does not significantly influence their depression. For older adults with a higher adaptation ability (1 standard deviation higher from mean), post-retirement work however increases their odd of depression, or in other words, staying in retirement decreases their odd of depression. This tests but does not support H3. These results are the opposite of our theoretical hypotheses. Figure 1 is a visual plot to illustrate the impact of post-retirement work on depression at different levels of adaptation ability. 

### 4.3. Robustness Check

In order to verify the robustness of the study results, we created a new dataset by randomly selecting another sample of 1433 non-working cases and ran the regression. With the new randomly selected non-working sample, the adaptation ability also significantly moderates the impact of post-retirement work on depression (B = 0.16, *p* < 0.01). Post-retirement work significantly decreases depression of older adults who are less adaptive (B = −0.11, *p* < 0.05) and significantly increases depression of older adults who are more adaptative (B = 0.12, *p* < 0.05). The results of the robustness check are consistent with the main study results. Additionally, we used the bootstrap technique to estimate the coefficients with multiple random samples and obtained similar results.

## 5. Discussion and Implications

Given the increasing popularity of post-retirement work and its demand for adaptation ability of older adults, this study examined the influence of post-retirement work on depression among Chinese older adults and the moderating effect of adaptation ability in the impact of post-retirement work on depression. The study reveals that, for less adaptive older adults, post-retirement work can prevent depression; however, for more adaptive older adults, staying in retirement instead of working may decrease the odds of depression. The results are just opposite with the theoretical hypotheses, yielding interesting theoretical and practical implications.

### 5.1. Theoretical Implications

The study hypotheses on the moderating role of adaptation ability were enlightened by the person–environmental fit theory, the trait activation theory, and the ecological theory of aging; however, the results are just interestingly the opposite of the theoretical hypotheses. The person–environmental fit theory indicates that post-retirement work may cause stress to the older adults who are not adaptive and in turn increase their depression. However, the result of the study demonstrates the beneficial effect of post-retirement work for this type of older adults. The trait activation theory posits the negative impact of post-retirement work on depression of more adaptive older adults, but the result shows a positive impact among this group. The surprising findings may be explained by the different traits and experiences between older adults and younger adults. For younger adults, their major life transitions are from campus to career or from previous job to a new job. Their major challenges exist in their new jobs; therefore, the person–environmental fit theory and the trait activation theory can explain how their adaptation ability play in work circumstances. That is to say that work may increase less adaptive workers’ depression and decrease more adaptive workers’ depression. Yet, to older adults, the challenges in retirement may be bigger than the challenges in post-retirement work. That is to say that retirement life requires a higher level of adaptation ability than post-retirement work does. Older adults with a higher adaptation ability can handle the challenging transition to retirement and enjoy retirement life so that their depression level could be much lower when they stay in retirement than when they work. However, older adults with a lower adaptation ability to handle the challenges of retirement have to rely on post-retirement work for continuity to avoid depression. These findings have threefold theoretical implications: first, they unveiled the deeper relationship between post-retirement work and depression. That is to say that post-retirement work does not always decrease depression. Second, they opened a new direction for post-retirement work research, suggesting the investigation of more constructs underlining the link between post-retirement work and mental health. Third, they demonstrated that a certain theory that explains younger adults’ psychology and behaviors well may not be applicable or even be opposite to older adults and thus called for future researchers to develop and test tailored theories for older adults.

Another possible explanation for the discrepancy between the results and the prediction could be the cultural difference. This study was conducted in China. Chinese culture is characterized by collectivism as a traditional value emphasizing reciprocity and loyalty [69,70]. There typically exists a strong tie between employees and their work [71], with which retirement transition could be more challenging for Chinese older adults than for western older adults. We therefore encourage future researchers to test the findings of the present study in Western countries.

### 5.2. Practical Implications

The results of the study demonstrate that post-retirement work cannot always prevent depression, and it depends on older adults’ adaptation ability. In order to maximize the benefit of post-retirement work, employers and social practitioners may consider the following implications.

First, as post-retirement work can prevent depression in less adaptive older adults, this type of old adults may be encouraged to work. Even though they are not adaptive and may be not able to meet work demand in the beginning, employers may offer training programs to facilitate their adaptation. For more adaptive older adults, instead of assuming that their adaptive ability would benefit their mental health in workplace, employers need to pay more attention to their mental health status and offer necessary consultancy if possible.

Second, since older adults with sufficient adaptation ability have lower possibility of depression in retirement than in post-retirement work, it is therefore not always a good idea to encourage post-retirement work for all older adults. For those who do not have to or want to work after retirement, it would be more feasible to help them improve their adaptation ability so as to maintain mentally healthy in retirement.

Third, in order to better analyze the discrepancy between the results and the prediction, Table 4 presents the demographic differences between the high and low adaptation groups along with Chi square result for each crosstab. The adaptation ability is significantly related to age, educational level, and neighborhood type. The number of differences between low and high adaptation ability was getting smaller as age increased. There were more low adaptive older adults in the literate group but more high adaptive older adults in other educational level groups. The number difference between low and high adaptation ability in rural older adults was smaller than that in urban older adults. These results suggest that age, educational level, and neighborhood type need to be considered when identifying older adults’ adaptation ability.

### 5.3. Limitations and Future Directions

Despite the unique theoretical and practical contributions, the study is not without limitations. First, the research dataset only includes a Chinese sample, as cultures may influence human psychology and behavior. Cross-cultural studies are needed to provide insightful cultural comparisons. Second, we conducted the research with one-off cross-sectional data, and information regarding respondents’ pre-retirement depression was not available. Future research could employ longitudinal or experimental designs to control pre-retirement mental health to test the robustness of our findings. Third, despite the merit of large sample size of the open dataset used for this study, some variables (e.g., type of work, level of pension, and pre-retirement work) that may benefit further investigation of the topic are not available. Future researchers collecting first-hand data may include these important variables in their study design to answer the related questions. Fourth, the study only examined adaptation ability as a conditional factor for the impact of post-retirement work. Future researchers are encouraged to investigate more possible conditional factors including factors relating to needs–supplies fit and value congruence to further reveal the deep relationship between post-retirement work and mental health.

## Figures and Tables

**Table 1:** Results of descriptive analysis.

						Non-Working		Working	
Characteristics	Category					n	%	n	%
Age	60–64					332	23.2%	589	41.1%
	65–69					391	27.3%	438	30.6%
	70–74					308	21.5%	203	14.2%
	75–79					201	14%	114	8%
	80 or above					201	14%	89	6.2%
Gender	Male					706	49.3%	854	59.6%
	Female					727	50.7%	578	40.4%
Marital Status	Married					985	68.7%	1178	82.2%
	Widowed					424	29.6%	237	16.5%
	Divorced					9	0.6%	11	0.8%
	Never married					15	1.0%	7	0.5%
Education	Illiterate					136	22.9%	88	12.4%
	Literate class					20	3.4%	33	4.7%
	Elementary school					212	35.8%	283	39.9%
	Middle school					153	25.8%	202	28.5%
	High/technical school					48	8.1%	86	12.1%
	College or above					24	4.0%	17	2.4%
Neighborhood	Rural					811	56.6%	844	58.9%
	Urban					622	43.4%	589	41.1%
		Non-working				Working			
	*p*	Min	Max	Mean	St. Deviation	Min	Max	Mean	St. Deviation
Health Status	0.00	1	5	3.30	0.95	1	5	3.54	0.91
Depression	0.04	1	3	1.70	0.37	1	3	1.67	0.38
Adaptation Ability	0.02	1	5	2.93	0.67	1	5	2.99	0.67

**Table 2:** Moderated regression analyses.

Independent Variables	B	SE	t	*p* Value
Constant	1.25 ***	0.24	5.06	0.00
Age	0.01 **	0.00	2.66	0.01
Gender	−0.08 **	0.03	−2.40	0.02
Marital Status	0.04	0.04	1.00	0.32
Education	0.05 ***	0.04	3.16	0.00
Neighborhood	−0.03	0.05	−0.65	0.52
Health Status	−0.02	0.02	−1.08	0.28
Pension	0.00	0.04	0.02	0.98
PRW	−0.54 ***	0.15	−3.65	0.00
AA	−0.04	0.04	−1.03	0.30
PRW × AA	0.19 ***	0.05	3.92	0.00
R^2^ = 0.09 ***	R^2^ change = 0.03 ***	F = 4.90 **	*p* = 0.00	

Note: PRW = post-retirement work; AA = adaptation ability, ** *p* < 0.05, *** *p* < 0.01.

**Table 3:** Conditional effects of post-retirement work on the values of adaptation ability.

Adaptation Ability	Effect	SE	t	*p*
M-1SD	−0.10 **	0.05	−2.16	0.03
M	0.02	0.03	0.70	0.48
M + 1SD	0.15 ***	0.05	3.21	0.00

Note: M = mean, SD = standard deviation, ** *p* < 0.05, *** *p* < 0.01.

**Table 4:** Crosstab of adaptation ability by respondents ‘characteristics.

		Low Adaptation Ability		High Adaptation Ability	
Characteristics	Category	n	%	n	%
Age	60–64	387	13.50%	534	18.63%
	65–69	366	12.77%	463	16.15%
	70–74	223	7.78%	288	10.05%
	75–79	169	5.90%	143	4.99%
	80 or above	130	4.54%	157	5.48%
Chi Square = 14.354		*p* = 0.006			
Gender	Male	695	24.25%	862	30.08%
	Female	580	20.24%	722	25.19%
Chi Square = 0.002		*p* = 0.961			
Marital Status	Married	954	33.29%	1206	42.08%
	Widowed	305	10.64%	353	12.32%
	Divorced	6	0.21%	14	0.49%
	Never married	10	0.35%	12	0.42%
Chi Square = 2.714		*p* = 0.438			
Education	Illiterate	131	4.57%	92	3.21%
	Literate class	21	0.73%	32	1.12%
	Elementary school	234	8.16%	259	9.04%
	Middle school	121	4.22%	234	8.16%
	High/technical school	39	1.36%	95	3.31%
	College or above	13	0.45%	27	0.94%
Chi Square = 50.656		*p* = 0.000			
Neighborhood	Rural	809	28.23%	845	29.48%
	Urban	466	16.26%	740	25.82%
Chi Square = 29.784		*p* = 0.000			
Work Status	Non-Working	663	23.13%	764	26.66%
	Working	612	21.35%	821	28.65%
Chi Square = 4.077		*p* = 0.043			

## Data Availability

Data can be found at class.ruc.edu.cn, accessed on 10 May 2023.
